# Post‐Diagnosis Hemorrhagic Events Are Strongly Associated With Poor Survival in Patients With Essential Thrombocythemia

**DOI:** 10.1002/jha2.70103

**Published:** 2025-07-15

**Authors:** Yoshinori Hashimoto, Tomoki Ito, Akihiko Gotoh, Mika Nakamae, Fumihiko Kimura, Michiaki Koike, Keita Kirito, Hideho Wada, Kensuke Usuki, Takayuki Tanaka, Takehiko Mori, Satoshi Wakita, Toshiki I. Saito, Akiko Kada, Akiko M. Saito, Kazuya Shimoda, Yuka Sugimoto, Toshiro Kurokawa, Akihiro Tomita, Yoko Edahiro, Hitoshi Kiyoi, Koichi Akashi, Itaru Matsumura, Katsuto Takenaka, Norio Komatsu

**Affiliations:** ^1^ Department of Hematology Tottori Prefectural Central Hospital Tottori Tottori Japan; ^2^ First Department of Internal Medicine Kansai Medical University Hirakata Osaka Japan; ^3^ Department of Hematology Tokyo Medical University Shinjuku‐ku Tokyo Japan; ^4^ Department of Hematology Osaka Metropolitan University Graduate School of Medicine Osaka Japan; ^5^ Division of Hematology National Defense Medical College Tokorozawa Saitama Japan; ^6^ Department of Hematology Juntendo University Shizuoka Hospital Izunokuni Shizuoka Japan; ^7^ Department of Hematology and Oncology University of Yamanashi Chuo‐Shi Yamanashi Japan; ^8^ Department of Hematology Kawasaki Medical School Kurashiki Okayama Japan; ^9^ Department of Hematology NTT Medical Center Tokyo Shinagawa Tokyo Japan; ^10^ Division of Hematology Keio University School of Medicine Shinjuku‐ku Tokyo Japan; ^11^ Department of Hematology Tokyo Medical and Dental University Bunkyo City Tokyo Japan; ^12^ Department of Hematology Nippon Medical School Bunkyo City Tokyo Japan; ^13^ Clinical Research Center NHO Nagoya Medical Center Nagoya Aichi Japan; ^14^ Division of Hematology Diabetes, and Endocrinology Department of Internal Medicine Faculty of Medicine University of Miyazaki Miyazaki Miyazaki Japan; ^15^ Department of Hematology and Oncology Mie University Tsu Mie Japan; ^16^ Department of Hematology Toyama Red Cross Hospital Toyama Japan; ^17^ Department of Hematology Fujita Health University School of Medicine Toyoake Aichi Japan; ^18^ Department of Hematology Juntendo University School of Medicine Bunkyo City Tokyo Japan; ^19^ Department of Advanced Hematology Juntendo University School of Medicine Bunkyo City Tokyo Japan; ^20^ Department of Hematology and Oncology Nagoya University Graduate School of Medicine Nagoya Aichi Japan; ^21^ Department of Medicine and Biosystemic Science Kyushu University Fukuoka Fukuoka Japan; ^22^ Department of Hematology and Rheumatology Faculty of Medicine Kindai University Higashiosaka Osaka Japan; ^23^ Department of Hematology Clinical Immunology and Infectious Diseases Ehime University Graduate School of Medicine Toon Ehime Japan; ^24^ PharmaEssentia Japan KK Minato‐ku Tokyo Japan

**Keywords:** essential thrombocythemia, hemorrhagic event, multistate Markov model, poor prognosis

## Abstract

**Objectives:**

The present study investigated the effects of thrombosis, hemorrhagic events, disease progression, and secondary malignancies on patient survival after the diagnosis of essential thrombocythemia (ET).

**Methods:**

We analyzed data from 1152 patients enrolled in the JSH‐MPN‐18 study using time‐dependent Cox regression and multistate Markov models to estimate transition hazards and state occupation probabilities.

**Results:**

Hemorrhagic events (hazard ratio = 2.92, 95% confidence interval = 1.78–4.78, *p* < 0.001) was associated with a poor prognosis. In multistate model, hazards from the hemorrhagic event to death were higher among cumulative transition hazards, and the probability of remaining in the hemorrhagic state was lower than the probability of remaining in the other states of thrombosis, disease progression, and secondary malignancy in state occupancy probabilities.

**Conclusions:**

The present results demonstrated that hemorrhagic events following the diagnosis of ET are a serious risk factor and are directly related to early death. Baseline characteristics and post‐diagnosis events (intermediate status) may both have a significant impact on survival, and treatment strategies that take into account the prevention of an intermediate status need to be incorporated into clinical practice.

**Trial Registration:**

The authors have confirmed clinical trial registration is not needed for this submission.

## 1 Introduction

The prognosis for patients diagnosed with essential thrombocythemia (ET) is not catastrophic; however, it is poorer than that of the general population due to the various events that may occur in its natural history, including thrombosis, hemorrhagic events, disease progression (myelofibrosis [MF] and acute myeloid leukemia), and secondary malignancies [1, 2]. Previous studies employed statistical methods to examine the effects of baseline characteristics at the time of the ET diagnosis on the time to onset of each of these events, and risk factors for each event have been identified [3, 4, 5, 6, 7]. However, real‐world scenarios are more complex, and ET may progress to death (absorbing state) after an event (intermediate state) during its follow‐up. Therefore, the investigation of death after an event is important for understanding which events may lead directly to death. The present study focused on the four most representative events stated above: thrombosis, hemorrhagic events, disease progression, and secondary malignancies. The impact of the development of each event on patient survival after the ET diagnosis was investigated.

## 2 Methods

This multicenter retrospective analysis was performed under the auspices of the Japanese Society of Hematology (JSH) to examine the clinical profiles of Japanese patients diagnosed with ET. We analyzed data from 1152 patients enrolled in the JSH‐MPN‐18 study, the largest cohort study of ET patients examined in Japan, aged 20 years or older, and were diagnosed with ET base on the 2008 or 2017 edition of the diagnostic criteria for the WHO classification [8, 9] between April 2005 and March 2018 [10]. With respect to thrombosis, each of the following conditions was individually classified: stroke, transient ischemic attack, myocardial infarction, angina pectoris, peripheral arterial occlusion, deep vein thrombosis, and pulmonary embolism. Conversely, splanchnic vein thrombosis, cerebral venous thrombosis, and other life‐threatening thrombosis were collectively classified as others. Cerebral hemorrhage, gastrointestinal hemorrhage, hematuria, mucosal hemorrhage, and others were categorized as hemorrhagic events. All thrombotic and bleeding events were collected as spontaneous events and did not include events related to trauma or surgery. Transformations were defined as cases that fulfilled each diagnostic criteria of the WHO classification 2017 [9], and secondary malignancies as new malignancies that had developed during the observation period regardless of the use of drugs.

In the initial analysis, a Cox proportional hazards model was employed to assess overall survival. The model included the baseline characteristics employed in the previous study (age of at least 60 years, sex, a white blood cell count of at least 11.0 × 10^9^/L, a platelet count of at least 1500 × 10^9^/L, a history of thrombosis, a history of hemorrhagic events, cardiovascular risk factors, and chromosomal abnormalities) [10], in addition to the occurrence of thrombosis, hemorrhagic events, disease progression, and secondary malignancy as time‐dependent variables. Multiple imputation by chained equations was performed for missing baseline characteristics. All continuous variables were dichotomized prior to analysis. The imputation model employed a logistic regression analysis, incorporating a range of demographic and clinical variables. This included age, sex, a white blood cell count of at least 11.0 × 10^9^/L, a platelet count of at least 1500 × 10^9^/L, a history of thrombosis, a history of hemorrhagic events, cardiovascular risk factors, and chromosomal abnormalities. Twenty imputations were generated using the fully conditional specification method. The analyses were performed using SAS version 9.4 (SAS Institute, Cary, NC).

In the second analysis, a multistate Markov model was used to estimate transition hazards through six states (ET diagnosis, thrombosis, hemorrhagic events, disease progression, secondary malignancy, and death) (Figure S1) [11]. To simplify the model, only the first event was used. Cumulative transition hazards were estimated by a nonparametric method, and state occupancy probabilities were calculated for the entire population. The same analysis was conducted for specific subgroups of major factors (age, white blood cell count, platelet count, *JAK2*V617F, history of thrombosis, and chromosomal abnormality). The present study was performed according to the principles of the Declaration of Helsinki, and was approved by the Ethics Committee of Tottori Prefectural Central Hospital (Approval No. 2023–65).

## 3 Results

The characteristics of the 1152 patients were as previously reported [10] and are shown in Table S1. The median observation period was 3.6 years (range: 0–15.4 years). The number of patients lost to follow‐up was 20. Following the ET diagnosis, thrombosis occurred in 75 patients, hemorrhagic events in 60, disease progression in 59, secondary malignancy in 36, and death in 81 (Table S2). Overall survival rates at 5 and 10 years were 93.6% (95% confidence interval (CI): 91.4%–95.2%), 5‐ and 10‐year thrombosis‐free survival rates were 92.7% (95% CI: 90.5%–94.3%) and 85.9% (95% CI: 81.4%–89.4%), and 5‐ and 10‐year bleeding event‐free survival rates were 93.9% (95% CI: 91.9%–95.4%) and 89.0% (95% CI: 85.2%–91.9%), respectively.

Events occurring after the second were also included in the initial analysis. After adjusting for baseline characteristics, hemorrhagic events (hazard ratio [HR] = 2.92, 95% CI = 1.78–4.78, *p* < 0.001) was associated with poor prognosis. HR of other events were thrombosis (HR = 1.03, 95% CI = 0.56–1.89, *p* = 0.918), disease progression (HR = 1.19, 95% CI = 0.70–2.01, *p* = 0.518), and secondary malignancy (HR = 1.56, 95% CI = 0.80–3.04, *p* = 0.191) (Table 1).

**TABLE 1:** Impact of thrombosis, hemorrhagic events, disease progression, and secondary malignancy on overall survival.

Variables	HR	95% CI	*p* value
Age ≥ 60 years	2.65	1.43–4.90	0.002
Sex (male)	1.01	0.62–1.66	0.956
WBC ≥ 11.0 × 10^9^/L	1.43	0.89–2.32	0.142
Plt ≥ 1500 × 10^9^/L	3.52	1.86–6.67	< 0.001
History of thrombosis	1.91	1.13–3.24	0.016
History of hemorrhagic events	2.95	1.27–6.86	0.012
Cardiovascular risk factors	1.51	0.83–2.76	0.181
Chromosome abnormality	2.06	0.89–4.80	0.093
Thrombosis	1.03	0.56–1.89	0.918
Hemorrhagic events	2.92	1.78–4.78	< 0.001
Disease progression	1.19	0.70–2.01	0.518
Secondary malignancy	1.56	0.80–3.04	0.191

*Note*: Time‐dependent variables were analyzed in a Cox proportional hazard model adjusted by age, sex, white blood cell count, platelet count, history of thrombosis, history of hemorrhagic events, cardiovascular risk factors, and chromosomal abnormalities.

Abbreviations: CI, confidence interval; HR, hazard ratio; Plt, platelets; WBC, white blood cells.

**FIGURE 1:**
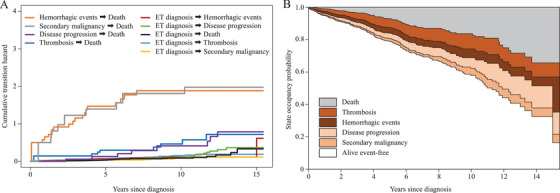
Cumulative transition hazard (A) and state occupancy probability (B) for state transitions. (A) Cumulative transition hazards are higher from the hemorrhagic events to death and from the secondary malignancy to death, followed by the hazards from thrombosis to death and from disease progression to death. (B) The probability of remaining in the hemorrhagic state is lower than the probability of remaining in the other event states.

In the second analysis, the first events included thrombosis in 68 patients, hemorrhagic events in 55, disease progression in 53, and secondary malignancy in 31, which differed from the number of events in the first analysis (Figure S1 and Table S2). In patients whose first event was thrombosis, a hemorrhagic event, disease progression, or secondary malignancy, 8, 18, 8, and 9, respectively, died (Figure S1). The first events and the direct cause of death did not always coincide, and details are provided in Table S3. Figure 1A,B show cumulative transition hazards and state occupancy probabilities for the entire population. Hazards from the hemorrhagic event to death and from the secondary malignancy to death were higher among cumulative transition hazards, followed by hazards from thrombosis to death and from disease progression to death. Consequently, the probability of remaining in the hemorrhagic state was lower than the probability of remaining in the thrombotic or disease progression states in state occupancy probabilities. This result implies that the occurrence of a hemorrhagic event shortens the time to death. Subgroup analyses of specific populations provided similar results (age < 60 years, age ≥ 60 years, white blood cell count < 11.0 × 10^9^/L, white blood cell count ≥ 11.0 × 10^9^/L, platelet count <1500 × 10^9^/L, platelet count ≥ 1500 × 10^9^/L, *JAK2*V617F‐positive, *JAK2*V617F‐negative, a history of thrombosis, no history of thrombosis, chromosomal abnormality, and no chromosomal abnormality) (data not shown). The treatment status at the time of each event occurrence is shown in Table S4. However, the impact of cytoreductive or antithrombotic drugs could not be accurately assessed due to missing treatment start dates.

## 4 Discussion

This is the first study to report the impact of post‐diagnostic events on the survival of ET patients in an Asian population. The results obtained demonstrated that hemorrhagic events are strong risk factor for death. Notably, hemorrhagic events had a high cumulative transition hazard to death and a low probability of state occupancy. Collectively, the present results indicate that hemorrhagic events, particularly intracranial hemorrhage, are likely to result in early death.

In a recent study, Carobbio et al. employed a multistate model, a novel approach for calculating the probabilities of multiple state transitions, and demonstrated that ET patients had a reduced probability of remaining in the state of disease progression, a higher mortality rate, and a shorter time to death [12]. This is consistent with the finding of an extremely poor treatment response in patients with post‐ET MF or blast phase myeloproliferative neoplasm (MPN‐BP) [13, 14]. The present results support recent findings and highlight the importance of intermediate states that are part of the natural history of MPN. A subsequent study on patients with polycythemia vera obtained similar findings, with an equivalent analytical approach indicating that the development of thrombosis and disease progression had a significant impact on mortality [15].

A previous review reported an incidence of hemorrhagic events of 12.5% (1.18/100 person‐years) overall and 4.57% (0.79/100 person‐years) for major hemorrhagic events [16]. The main sites of hemorrhage included the gastrointestinal tract, mucosa, and within the cranium. The mortality rate in cases of major hemorrhagic events was as high as 25% [17]. A recently reported retrospective European study of over 1300 patients showed that 10‐year survival was associated with hemorrhage, but not thrombosis, and that hemorrhage has a higher risk of mortality thrombosis [18]. In the JSH‐MPN‐R18 study [10], of the four major events occurring in patients with ET, hemorrhagic events were the most common cause of death (of all deaths, 17% were due to hemorrhagic events). In addition, the most frequent hemorrhagic sites were within the cranium and the gastrointestinal tract, as shown in Table S2. Table S3 shows that even in patients who developed thrombosis and then died, the direct cause was not necessarily thrombosis. However, in patients who died after a hemorrhagic event, the hemorrhagic event was the direct cause in more than 70% of patients. Hemorrhagic events should not be underestimated, particularly because intracranial hemorrhage negatively affects quality of life and leads to death in many cases.

Major hemorrhagic events, particularly an intracranial hemorrhage, may result in mortality within a relatively short time frame. By utilizing the multistate model, we further demonstrated that hemorrhagic events were more likely to result in mortality than other events. In addition, patients were less likely to remain in the state of hemorrhage for an extended period and were more likely to transition to death.

One of the limitations of the present study is that it was a retrospective cohort study; nondriver gene mutations and von Willebrand factor ristocetin cofactor activity were not included as a study measure, and the severity of hemorrhage was not defined. However, intracranial hemorrhage is considered to be equivalent to major bleeding as defined by the International Society on Thrombosis and Haemostasis [19], and the present result showing that intracranial hemorrhage (major bleeding) directly leads to death is significant (Table S3). The results of an ongoing prospective study (JSH‐MPN‐15) conducted by the Japanese Society of Hematology, which is paired with this study, will yield data regarding the severity of hemorrhage, prognosis, and the impact of treatments on post‐diagnosis events. Another limitation is that there were missing variables among baseline explanatory variables, which we addressed using multiple imputations. Furthermore, although this is a large‐scale cohort study of ET in Japan, the observation period is short. In this study, which had a relatively brief observation period, the cumulative transition hazard for bleeding events early after diagnosis was high. As previously indicated in the report [12], it is conceivable that the cumulative transition hazard for disease progression may increase with a longer observation period. This is an area of investigation in our ongoing prospective study. In addition, a multi‐state Markov model was used in the second analysis. This model does not adequately reflect clinical reality, and a model that allows for reversible transitions may better reflect clinical reality. In this study, the first event was used as the intermediate state in patients who experienced multiple events to simplify the model. However, the results obtained support the findings of a previous international collaborative study [12] and added new data beyond those regarding hemorrhagic events.

Although the frequency of secondary malignancies after the diagnosis of ET is not high, it is important to note that their occurrence is highly likely to result in death. In contrast to Europe and the United States, secondary malignancies in the JSH‐MPN‐R18 study were more likely to be gastrointestinal cancers, suggesting the effects of ethnic differences [10]. Therefore, after the diagnosis of ET, it is important to focus not only on the control of blood cell counts, but also on the development of solid cancers.

In conclusion, the present study demonstrated that hemorrhagic events following the diagnosis of ET are a similar or more serious risk factor than other representative events and are directly related to death. Baseline characteristics and the intermediate status may both have a significant impact on survival, and treatment strategies that take into account the prevention of an intermediate status need to be incorporated into clinical practice in the future.

## Author Contributions

Y.H. and N.K. planned the study and Y.H. wrote the manuscript. A.K. performed the statistical analysis and contributed to manuscript writing. T.I., A.G., M.N., F.K., M.K., K.K., H.W., K.U., T.T., T.M., S.W., T.I.S., A.M.S., K.S., Y.S., T.K., A.T., Y.E., H.K., K.A., I.M., K.T., and N.K. collected data and helped with manuscript writing. All authors revised and approved the final version of the manuscript.

## Ethics Statement

The study was approved by the Ethics Committee of Tottori Prefectural Central Hospital (Approval No. 2023–65).

## Consent

The study data were analyzed anonymously and the patient privacy was protected. The patients were assured that participation was voluntary and that they may opt out of information disclosure. Written informed consent requirements were waived for this study by the Ethics Committee.

## Conflicts of Interest

The authors declare no conflicts of interest.

## Supporting information




**Table s1**:Patient characteristics**Table s2**:Details of post‐diagnosis events**Table s3**:Details on the direct cause of death after each first event**Table s4**:Details on cytoreductive therapies and antiplatelet and anticoagulant therapies at the time of each event occurrence.

**Supporting File 2: Supplementary Figure 1**: Outline of the six‐states model for the clinical course of patients with ET. The six states, represented by boxes, are the ET diagnosis, thrombosis, hemorrhagic events, disease progression, secondary malignancy, and death. The number of patients who initiated (on the left) and completed (on the right) in each state is shown within the respective boxes. Arrows indicate the number of patients involved in the corresponding transition.

## Data Availability

The datasets generated and/or analyzed during the present study are available from the corresponding author upon reasonable request.
