# Supplementary material for: Post‐Diagnosis Hemorrhagic Events Are Strongly Associated With Poor Survival in Patients With Essential Thrombocythemia
Source: EJHaem. 2025 Jul 15;6(4):e70103. doi: 10.1002/jha2.70103 (PMC12260263; doi:10.1002/jha2.70103)
Supplement: Supplementary file 1 — Table s1:Patient characteristicsTable s2:Details of post‐diagnosis eventsTable s3:Details on the direct cause of death after each first eventTable s4:Details on cytoreductive therapies and antiplatelet and anticoagulant therapies at the time of each event occurrence. [file JHA2-6-e70103-s002.docx]

**Supplementary Table 1** Patient characteristics

| Characteristics at diagnosis | Missing, n (%) ^$^ | Proportion, % (SE) ^†^ |
| --- | --- | --- |
| Age ≥60 years | 0 (0) | 60.4 (1.4) |
| Male | 0 (0) | 44.5 (1.5) |
| WBC ≥11.0×10^9^/L | 4 (0.3) | 32.5 (1.4) |
| Plt ≥1,500×10^9^/L | 3 (0.3) | 7.1 (0.8) |
| History of thrombosis | 17 (1.5) | 18.2 (1.2) |
| History of hemorrhagic events | 20 (1.7) | 2.2 (0.4) |
| Cardiovascular risk factors ^#^ | 163 (14.1) | 62.4 (1.6) |
| Chromosome abnormality | 143 (12.4) | 9.1 (0.9) |

^#^ Cardiovascular risk factors were defined as cases with at least one of hypertension, diabetes mellitus, hyper-low-density lipoprotein cholesterolemia, hypertriglyceridemia and/or current smoking.

**^$^** Missing among 1152 patients.

**^†^** Proportion and SE were summarized from 20 imputed data.

*n*, number of patients; *Plt*, platelets; *SE*, standard error; *WBC*, white blood cells

**Supplementary Table 2** Details of post-diagnosis events

| Each event and its details | Number of patients |
| --- | --- |
| **Thrombotic events, n (%)** | **75 (6.5)** |
| Stroke, n (%) | 33 (2.9) |
| Transient ischemic attack, n (%) | 7 (0.6) |
| Myocardial infarction, n (%) | 9 (0.8) |
| Angina pectoris, n (%) | 8 (0.7) |
| Peripheral arterial occlusive disease, n (%) | 4 (0.3) |
| Deep vein thrombosis, n (%) | 3 (0.3) |
| Pulmonary embolism, n (%) | 3 (0.3) |
| Others, n (%) | 8 (0.7) |
| **Hemorrhagic events, n (%)** | **60 (5.2)** |
| Cerebral hemorrhage, n (%) | 18 (1.6) |
| Gastrointestinal hemorrhage, n (%) | 18 (1.6) |
| Hematuria, n (%) | 4 (0.3) |
| Mucosal hemorrhage (oral cavity), n (%) | 4 (0.3) |
| Mucosal hemorrhage (nasal mucosa), n (%) | 10 (0.9) |
| Others, n (%) | 6 (0.5) |
| **Disease progression, n (%)** | **59 (5.1)** |
| Acute leukemia, n (%)^#^ | 11 (1.0) |
| Myelodysplastic syndromes, n (%) | 5 (0.4) |
| Secondary myelofibrosis, n (%)^#^ | 34 (3.0) |
| Polycythemia vera, n (%) | 10 (0.9) |
| Others, n (%) | 1 (0.1) |
| **Secondary malignancies, n (%)** | **36 (3.1)** |
| Gastrointestinal cancer, n (%) | 9 (0.8) |
| Lymphoma, n (%)^$^ | 7 (0.6) |
| Urological cancer, n (%)^$^ | 4 (0.3) |
| Lung cancer, n (%) | 3 (0.3) |
| Non-melanoma skin cancer, n (%) | 1 (0.1) |
| Thyroid cancer, n (%) | 1 (0.1) |
| Gastrointestinal stromal tumor, n ‘%) | 1 (0.1) |
| Splenic angiosarcoma, n (%) | 1 (0.1) |
| Chronic myeloid leukemia, n (%) | 1 (0.1) |
| Others, n (%)^$^ | 10 (0.9) |
| **Death, n (%)** | **81 (7.0)** |
| Thrombotic events, n (%) | 4 (0.3) |
| Hemorrhagic events, n (%) | 14 (1.2) |
| Disease progression, n (%) | 7 (0.6) |
| Secondary malignancies, n (%) | 7 (0.6) |
| Infections, n (%) | 11 (1.0) |
| Heart failure due to causes other than those listed above, n (%) | 19 (1.6) |
| Unknown, n (%) | 15 (1.3) |
| Others, n (%) | 4 (0.3) |

^#^Two patients were included in both acute leukemia and secondary myelofibrosis.

^$^One patient had lymphoma and urological cancer; the other had two other malignancies.

**Supplementary Table 3** Details on the direct cause of death after each first event

| Cause of death | Thrombosis  Number of deaths: 8 | Hemorrhagic events  Number of deaths: 18 | Disease progression  Number of deaths: 8 | Secondary malignancy  Number of deaths: 9 |
| --- | --- | --- | --- | --- |
| Thrombosis | **2** | 1 | 0 | 1 |
| Cerebral hemorrhage | 1 | **11** | 0 | 0 |
| Gastrointestinal hemorrhage | 0 | **1** | 0 | 0 |
| Other hemorrhagic events | 0 | **1** | 0 | 0 |
| Disease progression | 0 | 2 | **4** | 0 |
| Secondary malignancies | 0 | 1 | 0 | **6** |
| Infections | 1 | 0 | 3 | 0 |
| Other causes | 3 | 1 | 0 | 1 |
| Unknown | 1 | 0 | 1 | 1 |

Patients whose first event and direct cause of death were the same are shown in bold.

**Supplementary Table 4** Details on cytoreductive therapies and antiplatelet and anticoagulant therapies at the time of each event occurrence

| Treatment | Thrombosis  n=75 | Hemorrhagic events n=60 | Disease progression  n=59 | Secondary malignancy  n=36 |
| --- | --- | --- | --- | --- |
| Cytoreductive therapies, n | **37** | **34** | **39** | **28** |
| HU only, n | 16 | 21 | 17 | 22 |
| ANA only, n | 15 | 11 | 10 | 3 |
| Combination of HU and ANA, n | 6 | 2 | 5 | 2 |
| Other agents, n | 0 | 0 | 7 | 1 |
| None, n | 37 | 25 | 20 | 8 |
| Unknown, n | 1 | 1 | 0 | 0 |
| Antiplatelet and anticoagulant therapies, n | **36** | **35** | **23** | **21** |
| Antiplatelet agents only, n | 25 | 30 | 23 | 20 |
| Anticoagulants only, n | 7 | 2 | 0 | 0 |
| Combination of antiplatelet and anticoagulant drugs, n | 3 | 3 | 0 | 1 |
| Other agents, n | 1 | 0 | 0 | 0 |
| None, n | 38 | 24 | 36 | 15 |
| Unknown, n | 1 | 1 | 0 | 0 |

The total number of patients receiving each cytoreductive therapy and the total number of patients receiving each antiplatelet agent and anticoagulant are shown in bold.

*ANA*, anagrelide; *HU*, hydroxyurea; *n*, number of patients
