# Supplementary figures and images for: Post‐Diagnosis Hemorrhagic Events Are Strongly Associated With Poor Survival in Patients With Essential Thrombocythemia
Source: EJHaem. 2025 Jul 15;6(4):e70103. doi: 10.1002/jha2.70103 (PMC12260263; doi:10.1002/jha2.70103)

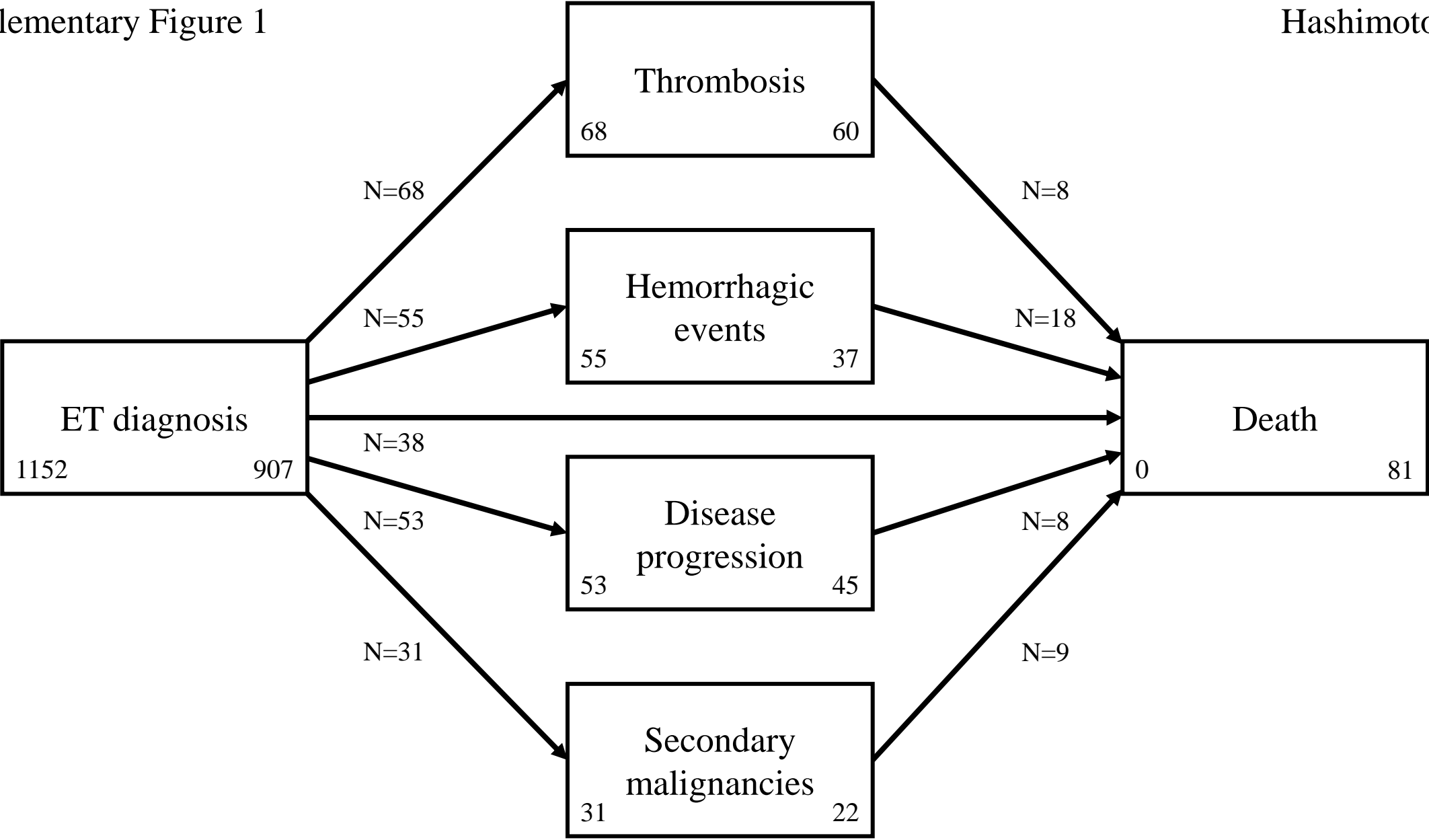

Supplement: Supplementary file 2 — Supporting File 2: Supplementary Figure 1: Outline of the six‐states model for the clinical course of patients with ET. The six states, represented by boxes, are the ET diagnosis, thrombosis, hemorrhagic events, disease progression, secondary malignancy, and death. The number of patients who initiated (on the left) and completed (on the right) in each state is shown within the respective boxes. Arrows indicate the number of patients involved in the corresponding transition. [file JHA2-6-e70103-s001.pdf]
